# Media representations of COVID-19 public health policies: assessing the portrayal of essential health services in Canadian print media

**DOI:** 10.1186/s12889-021-10300-2

**Published:** 2021-02-03

**Authors:** Ubaka Ogbogu, Lorian Hardcastle

**Affiliations:** 1 Faculties of Law and Pharmacy and Pharmaceutical Sciences, University of Alberta, Law Centre, 111 St and 89 Avenue, Edmonton, Alberta T6G 2H5 Canada; 2 Faculty of Law and Cumming School of Medicine, University of Calgary, 2500 University Drive NW, Calgary, Alberta T2N 1N4 Canada

**Keywords:** COVID-19, Public health, Public health policy, Media representations of health, Public understanding of health, Essential services, Liquor and cannabis sales

## Abstract

**Aims:**

The study assessed how the Canadian print media represented essential healthcare services during the COVID-19 pandemic, including the controversial decision to include liquor and cannabis stores in essential services lists.

**Methods:**

Mixed-method content analysis of 67 articles published in major Canadian English language newspapers between March 23 and April 1, 2020. Articles were analyzed and coded by two raters. Ratings were analyzed in SPSS.

**Results:**

Few articles in the sample discussed essential healthcare services and the inclusion of liquor and cannabis stores in essential services lists. Majority of the articles that discussed both topics framed the discussion positively and consistently with current knowledge and evidence.

**Conclusion:**

Canadian print media representations of essential healthcare services and associated public debate are largely descriptive and, therefore, fail to engage critically with or advance public understanding of an important health policy issue.

**Supplementary Information:**

The online version contains supplementary material available at 10.1186/s12889-021-10300-2.

## Background

Closing non-essential services was one of the measures deployed for dealing with the COVID-19 pandemic. Essential services are those “considered critical to preserving life, health, public safety and basic societal functioning” [1]. In the context of a pandemic, this term refers to various undertakings that are allowed to remain open despite a public health order requiring the closure of businesses and services due to a public health emergency. Common examples of essential services include urgent healthcare, policing, grocery stores, and transportation/delivery. However, what is considered essential also depends on local social, political, and economic contexts. For example, the inclusion of the energy sector in Alberta’s list of essential services reflects the importance of the sector to the province’s economy [2].

The Ontario government released the first list of essential services in Canada on March 23, 2020 [3]. The list, which included 19 sectors deemed essential, was criticized by some for including beer, wine and liquor stores, cannabis stores, and alcohol and cannabis producers. In response, Ontario subsequently delisted cannabis stores and producers, although cannabis is still available through the Ontario government's online retail outlets [4, 5]. Similar criticisms arose when other provinces decided to include or exclude cannabis from the list of essential services or to limit access to these products (e.g. making cannabis available via online delivery only and not in retail outlets) [6, 7].

Public opinion of alcohol and cannabis diverge from how some health policy experts view their inclusion on essential services lists. These experts contend that there are valid health-related reasons for keeping these businesses open [8–10]. Individuals who are addicted to alcohol can experience serious medical conditions including seizure and death, or may switch to consuming more harmful products that contain alcohol such as rubbing alcohol, mouthwash, or even gasoline [10]. Keeping liquor supplies open can help ensure that alcohol-dependent persons do not suffer withdrawal or consume unsafe products and seek access to emergency medical care at a time when hospitals are focused on COVID-19. Given that heavy alcohol use can affect the immune system and make individuals more vulnerable to respiratory disease [11–13], it can be particularly dangerous for them to access hospital services. Allowing liquor stores to remain open can also be viewed as a means of maintaining system-wide integrity during a pandemic by avoiding a situation where the decision to close liquor stores results in a spike in demand for healthcare services such as addiction and mental health supports.

There are also several health-related arguments for leaving cannabis stores open during a pandemic. Retail cannabis stores are subject to licensing and operational requirements that ensure quality control, ease of access, and education regarding safe use. Consumers would be better protected by purchasing cannabis from stores that are subject to these requirements than from non-regulated sources. The products available in retail outlets may also be safer than those on the black market, which are not subject to the same testing or labelling requirements, may be more potent than what the consumer is used to, or may be laced with unknown substances. In addition, with the decriminalization of recreational cannabis, some medical users have shifted to buying cannabis without first obtaining a medical document [14]. If recreational stores were closed, medical users may be compelled to obtain a medical document to continue treating their condition, at a time when access to physicians may be limited.

The argument that liquor and cannabis sales can help maintain health system integrity during a pandemic is contested [4]. Those seeking closure of these businesses contend that persons who abuse substances or suffer from addiction are likely to violate public health directives such as physical distancing [4, 15] and may increase the strain on healthcare resources by seeking treatment services during the pandemic [16]. Increased alcohol intake during the pandemic can also lead to a crisis of new or increased addiction disorders at serious costs to the healthcare system [17]. This increased intake may also cause a rise in other health-related risks such as domestic violence or accidental injuries [18].

It is well established that the mass media plays important roles in policy making [19, 20]. The functions served by the mass media in the policy making process include shaping and influencing public opinion and simplifying and amplifying policy messages for public consumption [19, 20]. While the media can influence public opinion on health-related matters, the role that it plays in portraying controversial public health issues, especially in the context of a pandemic, is less well understood. This study aims to fill this gap by examining media portrayal of controversial aspects of policies delineating essential health services during the COVID-19 pandemic, with particular focus on the controversy over the inclusion of alcohol and liquor sales in essential services lists.

## Methods

We searched Factiva™ for terms used to describe essential services in provincial/territorial and federal policies. Factiva™ is an international news aggregator and database. The search string was [“essential service” or “essential business” or “allowable business” or “priority service” or “essential workplace”]*.* Sources selected for the search were major Canadian English language newspapers. French language newspapers were excluded due to non-availability of raters proficient in French. The search was restricted to articles published between 23 March 2020, when the Ontario government released the first essential services list in Canada, and April 1, 2020, when the search was conducted.

The search yielded an initial sample of 229 articles. Duplicates and non-media press releases were excluded for a final sample of 67 articles. Two raters analyzed the articles using a coding frame derived from published peer-reviewed studies (see Supplementary Material S1 for the coding frame). Rater observations were recorded on an Excel spreadsheet by assigning numerical values to the variables in the coding frame. Although responses were reported numerically, some of the variables necessitated a qualitative, interpretative analysis of the articles and selecting a pre-determined response that best matched the rater’s analysis and interpretation. The first rater analyzed the full sample while the second rater reviewed a subset for purposes of inter-rater reliability. Inter-rater agreement was tested using Cohen’s K. There was substantial agreement on two variables, moderate agreement on four, and no agreement on three variables (questions 5–7 in the Coding Frame S1). Disagreements between the raters were resolved via discussion to reach a consensus [21, 22] and the entire sample was re-analyzed based on interpretations agreed on by both raters. The final dataset was analyzed in SPSS™, a statistical analysis software that is used to perform descriptive statistics, including frequencies and cross-tabulations, on assigned values (see Supplementary Material S2 for the raw data). Cross-tabulations were analyzed using the Fisher’s Exact two-sided test.

## Results

### Type and author of articles, and jurisdictions discussed

The study sample included 61 news articles (91%) and 6 articles from the editorial and opinion section (9%). The majority of the articles were written by journalists or reporters (62 articles; 92.52%). Of the remaining 5 articles, 2 (3%) were written by non-media experts and 3 (4.5%) did not specify the authors.

The jurisdictions discussed in the articles include Ontario (20 articles, 29.9%), British Columbia (15 articles, 22.4%), Alberta and Quebec (4 articles each, 6% respectively), Nova Scotia (3 articles, 4.5%) and Saskatchewan (2 articles, 3%). Nineteen articles (28.4%) discussed multiple jurisdictions.

### Healthcare as an essential service

The raters assessed whether and in what manner the articles *discussed* healthcare as an essential service. Articles that simply mentioned or listed healthcare services that were included in the essential services list without any further discussion were not included in the ratings.

Only 2 articles (3%) exclusively discussed healthcare as an essential services sector. Five articles (7.5%) discussed multiple sectors including healthcare, while 7 articles (10.4%) discussed multiple sectors but did not include healthcare in the discussion. The majority of the articles (44, 65.7%) discussed a single sector other than healthcare, while 9 articles (13.4%) did not mention or discuss any sector.

All the articles that discussed healthcare (either exclusively or as part of a discussion of multiple sectors) (*n* = 7, 10.4%) discussed healthcare services other than diagnostic and treatment services aimed at achieving a cure or managing symptoms. Based on the coding frame, healthcare services other than diagnostic or treatment services include preventative care services (such as vaccinations) and health promotion and improvement services (such as gyms). All seven articles were supportive of including healthcare services other than diagnostic and treatment services in the essential services list, i.e. viewed the inclusion of these services in the list in positive terms.

### Liquor and cannabis stores as essential services

Raters recorded articles that *discussed* the inclusion of liquor and cannabis stores in the essential services lists. For this rating, articles that merely mentioned or listed liquor and cannabis stores were not included. Only a few articles in the sample (6 articles, 9%) discussed the inclusion of liquor and cannabis stores in essential services lists. All 6 articles also stated or implied that liquor and cannabis stores were essential services. Three of the 6 articles that discussed liquor and cannabis stores were supportive of including liquor and cannabis stores in the essential services lists. None of the articles in the sample were critical of including liquor and cannabis stores in the lists.

### Commentaries on healthcare and inclusion of liquor and cannabis stores

Since commentaries are largely opinion-based rather than reportage, we sought to assess general trends in the discussions in the commentaries. Two of the six opinion/editorial articles in the sample discussed healthcare as an essential services sector (*p = .209*). The same number of opinion/editorial articles also framed healthcare broadly to include healthcare services other than diagnostic or treatment services (*p = .115*) and supported including these other services in the essential services list (*p = .115*).

Only one opinion/editorial discussed liquor and cannabis stores (*p = .444 0.001*). This article stated or implied that liquor and cannabis stores were essential services (*p = .444*) and was supportive of including them in the list of essential services (*p = .304*). None of the articles that discussed healthcare and/or liquor and cannabis stores were written by experts.

## Discussion

The study findings have modest but important implications for our understanding of the role that the print media plays in framing public policy and debate during a public health crisis such as the COVID-19 pandemic.

The most significant finding is that print media reporting on the essential services debate was largely descriptive and uncritical. Most of the articles focused on the reporting of facts (such as which services had been labelled essential in which provinces) without deeper discussion or framing of the factors that go into determining whether something is an essential service. To that end, the media largely did not pick up on the essential services debate as it relates to either healthcare services or liquor/cannabis stores. Interestingly, the few opinion/editorial pieces that did address these issues raised the debate over healthcare and liquor/cannabis as essential services (compared to the portion of news articles that addressed this debate).

The nature of COVID-19, which created significant public pressure to immediately release facts as soon as they became available and to keep pace with the news cycle, may have disincentivized more exploratory journalism. Studies of media reporting in other contexts have found or a connection between expedient news production and less critical/cautious reporting [23–26]. Media concentration and news syndication – prominent features of the Canadian print media landscape – may also be a factor. A majority of Canadian English language newspapers are owned by one corporation, and the national news syndication agency, the Canadian Press, is jointly-owned by three of Canada’s largest media corporations. It has been noted that media concentration and syndication may result in homogenization of journalistic practices and adversely affect the diversity and quality of reporting [24, 27].

The findings also suggest a disconnect between public and media discourse. While journalists were silent or not critical of the inclusion of liquor and cannabis stores as essential services, members of the public criticized this decision as being financially motivated (which is not unwarranted, given that some provinces framed the inclusion of liquor and cannabis, at least in part, as an economic issue) [28]. When public discourse was not critical, it tended to conceptualize liquor and cannabis not as healthcare services but as leisure pursuits [29]. Even Quebec’s premier, Francois Legault, offered justifications for keeping liquor stores open that seemed to trivialize the complexity of liquor consumption during the pandemic when he stated “[t]o reduce the stress, you have to do some exercise...but sometimes a glass of wine may help” [29]. Criticism of the decision also emerged in the media well after the lists of essential services had been determined, such as those by some First Nations groups concerned about increased alcohol consumption during the pandemic [10].

While none of the articles that discussed liquor and cannabis stores were written by experts, our review of articles that reported on the issue indicates that expert opinion was sought and referenced by journalists. Thus, print media coverage of the issue, while not substantial, was informed by expert commentary. Given the role of syndication in Canadian print media (our initial search yielded a large number of duplicates), it is safe to include that such expert commentary is also widely dispersed. Still, the lack of expert-driven commentary suggests that experts had limited contribution to print media discourse on an important public policy debate.

One limited but important finding is that articles that discussed healthcare framed essential services within the sector robustly to include services other than diagnostic and treatment services, such as health promotion and preventative care services. This framing suggests an understanding of health as “a state of complete physical, mental, and social well-being and not merely the absence of disease or infirmity” [30]. It also emphasizes the team-based and holistic nature of healthcare. Keeping health promotion and preventative care services open during a pandemic can prevent health effects that drive people to seek treatment services, thus helping to maintain the integrity of the health system as a whole. It has been reported, for example, that childhood vaccination rates have declined since the pandemic began due to shelter-in-place policies, limitations on movement of goods, and other interventions aimed at addressing the pandemic [31–33]. This situation would be made worse by limiting or closing vaccination centres. Indeed, an argument could be made for home delivery of vaccination services to prevent a re-emergence of childhood infectious diseases during or after the pandemic.

The non-inclusion of French language print media is a limitation of this study. Inclusion may have yielded different results and influenced our analysis.

## Conclusion

The decision to include liquor and cannabis sales on essential services lists generated public criticism for being motivated by tax-revenue or linked to an increased risk of domestic violence and substance abuse. These criticisms diverge from expert opinion, which largely supports the availability of these products for health reasons. The media has an important role to play in providing information in a manner that mediates this gap between public and expert opinion. Better public understanding of the rationales underlying governmental policy choices improves the quality of democratic debate, which is particularly important during a pandemic, where governments tend to make decisions that intrude on individual rights. In the context of essential services, print media tended to inform the public about which services would close and which would remain open, rather than engaging in a discussion of their merits (either health-related or otherwise). However, where they did engage in deeper analysis, print media representations of essential healthcare services were consistent with current expert-generated knowledge and evidence.

## Supplementary Information

**Additional file 1.** Coding Frame.

**Additional file 2.** Raw Data.

## Data Availability

All data generated or analyzed during this study are included in this published article [and its supplementary information files].
