# Supplementary material for: Media representations of COVID-19 public health policies: assessing the portrayal of essential health services in Canadian print media
Source: BMC Public Health. 2021 Feb 3;21:273. doi: 10.1186/s12889-021-10300-2 (PMC7856611; doi:10.1186/s12889-021-10300-2)
Supplement: Supplementary file 1 — Additional file 1. Coding Frame. [file 12889_2021_10300_MOESM1_ESM.pdf]

Enter numbers attached to each code in the excel file (numbers only please). Please insert all descriptive codes in a separate column next to the associated variable (e.g. where asked to specify "other").

1) Article type
    a) News article – 1
    b) Editorial or opinion – 2
    c) Other (specify) – 3
    d) Not specified or unclear – 4

2) Author or article source?
    a) Journalist/Reporter – 1
    b) Expert – 2
    c) Other (specify) – 3
    d) Not specified or unclear – 4

3) Jurisdiction(s) discussed in the article, i.e. source of the essential services list discussed in the article:
    a) Alberta – 1
    b) BC – 2
    c) Manitoba – 3
    d) New Brunswick – 4
    e) Newfoundland & Labrador – 5
    f) Northwest Territories - 6
    g) Nova Scotia – 7
    h) Nunavut – 8
    i) Ontario – 9
    j) Prince Edward Island – 10
    k) Saskatchewan – 11
    l) Quebec – 12
    m) Yukon – 13
    n) More than one jurisdiction – 14

4) What sectors are discussed in the article? (do not include mere mentions)
    a) Multiple sectors – 1
    b) Multiple sectors, including healthcare - 2
    c) Healthcare only – 3
    d) One other sector only – 4
    e) No sectors mentioned or discussed – 5

5) Does the article discuss healthcare sectors other diagnostic and treatment services aimed at achieving a cure or managing symptoms? Other healthcare sectors includes preventative care (e.g. vaccinations), health promotion or health improvement services (e.g. gyms):
    a) Yes – 1
    b) No – 2

6) If other healthcare sectors are discussed, does the article support including them in the list of essential services:
    a) Yes – 1
    b) No – 2
    c) Not applicable or does not include a view or opinion on whether these sectors should be included – 3

7) Are liquor stores and/or cannabis stores discussed? (do not include mere mentions)
    a) Yes – 1
    b) No – 2

8) Does the article state or imply that liquor stores and/or cannabis stores are essential health services?
    a) Yes – 1
    b) No – 2

9) If liquor and/or cannabis stores are discussed in the article, is the article:
   a) Supportive of including them in the list of essential services – 1
   b) Critical of including them in the list of essential services – 2
   c) Neutral or balanced regarding including them in the list of essential services – 3
   d) Silent on including them in the list of essential services – 4
